# Supplementary material for: COZOID: contact zone identifier for visual analysis of protein-protein interactions
Source: BMC Bioinformatics. 2018 Apr 6;19:125. doi: 10.1186/s12859-018-2113-6 (PMC5889581; doi:10.1186/s12859-018-2113-6)
Supplement: Supplementary file 2 — Software build. Executable binary file of the software tool. (ZIP 118,784 kb) [file 12859_2018_2113_MOESM2_ESM.zip › cozoid/java/docs/beansbinding-1.2.1-doc.zip!/org/jdesktop/el/impl/util/ReflectionUtil.html]

ReflectionUtil (Beans Binding)


---


|  |  |  |
| --- | --- | --- |
| **Overview** \| **Package** \| **Class** \| **Use** \| **Tree** \| **Deprecated** \| **Index** \| **Help** | |  |
| **PREV CLASS**   NEXT CLASS | **FRAMES**    **NO FRAMES**     **All Classes** |  |
| SUMMARY: NESTED \| FIELD \| CONSTR \| METHOD | DETAIL: FIELD \| CONSTR \| METHOD |  |


---


## org.jdesktop.el.impl.util Class ReflectionUtil

```
java.lang.Object
  org.jdesktop.el.impl.util.ReflectionUtil
```

---

``` public class ReflectionUtil extends java.lang.Object ```

Utilities for Managing Serialization and Reflection

---

| **Field Summary** | |
| --- | --- |
| `protected static java.lang.String[]` | `EMPTY_STRING` |
| `protected static java.lang.String[]` | `PRIMITIVE_NAMES` |
| `protected static java.lang.Class[]` | `PRIMITIVES` |


| **Method Summary** | |
| --- | --- |
| `static java.lang.Class` | `forName(java.lang.String name)` |
| `protected static java.lang.Class` | `forNamePrimitive(java.lang.String name)` |
| `static java.lang.reflect.Method` | `getMethod(java.lang.Object base, java.lang.Object property, java.lang.Class[] paramTypes)`             Returns a method based on the criteria |
| `static java.beans.PropertyDescriptor` | `getPropertyDescriptor(java.lang.Object base, java.lang.Object property)` |
| `protected static java.lang.String` | `paramString(java.lang.Class[] types)` |
| `static java.lang.Class[]` | `toTypeArray(java.lang.String[] s)`             Converts an array of Class names to Class types |
| `static java.lang.String[]` | `toTypeNameArray(java.lang.Class[] c)`             Converts an array of Class types to Class names |

**Methods inherited from class java.lang.Object**

`clone, equals, finalize, getClass, hashCode, notify, notifyAll, toString, wait, wait, wait`

**Field Detail**

### EMPTY\_STRING

```
protected static final java.lang.String[] EMPTY_STRING
```

---


### PRIMITIVE\_NAMES

```
protected static final java.lang.String[] PRIMITIVE_NAMES
```

---


### PRIMITIVES

```
protected static final java.lang.Class[] PRIMITIVES
```


**Method Detail**

### forName

```
public static java.lang.Class forName(java.lang.String name)
                               throws java.lang.ClassNotFoundException
```

:   **Throws:**: `java.lang.ClassNotFoundException`

---


### forNamePrimitive

```
protected static java.lang.Class forNamePrimitive(java.lang.String name)
```

---


### toTypeArray

```
public static java.lang.Class[] toTypeArray(java.lang.String[] s)
                                     throws java.lang.ClassNotFoundException
```

:   Converts an array of Class names to Class types

    :   **Parameters:**: `s` - **Returns:**: **Throws:**: `java.lang.ClassNotFoundException`

---


### toTypeNameArray

```
public static java.lang.String[] toTypeNameArray(java.lang.Class[] c)
```

:   Converts an array of Class types to Class names

    :   **Parameters:**: `c` - **Returns:**

---


### getMethod

```
public static java.lang.reflect.Method getMethod(java.lang.Object base,
                                                 java.lang.Object property,
                                                 java.lang.Class[] paramTypes)
                                          throws MethodNotFoundException
```

:   Returns a method based on the criteria

    :   **Parameters:**: `base` - the object that owns the method: `property` - the name of the method: `paramTypes` - the parameter types to use **Returns:**: the method specified **Throws:**: `MethodNotFoundException`

---


### paramString

```
protected static final java.lang.String paramString(java.lang.Class[] types)
```

---


### getPropertyDescriptor

```
public static java.beans.PropertyDescriptor getPropertyDescriptor(java.lang.Object base,
                                                                  java.lang.Object property)
                                                           throws ELException,
                                                                  PropertyNotFoundException
```

:   **Parameters:**: `base` -: `property` - **Returns:**: **Throws:**: `ELException`: `PropertyNotFoundException`


---


|  |  |  |
| --- | --- | --- |
| **Overview** \| **Package** \| **Class** \| **Use** \| **Tree** \| **Deprecated** \| **Index** \| **Help** | |  |
| **PREV CLASS**   NEXT CLASS | **FRAMES**    **NO FRAMES**     **All Classes** |  |
| SUMMARY: NESTED \| FIELD \| CONSTR \| METHOD | DETAIL: FIELD \| CONSTR \| METHOD |  |


---
